# Acute kidney injury and mortality risk in older adults with COVID-19

**DOI:** 10.1007/s40620-021-01022-0

**Published:** 2021-03-22

**Authors:** Hong Xu, Sara Garcia-Ptacek, Martin Annetorp, Annette Bruchfeld, Tommy Cederholm, Peter Johnson, Miia Kivipelto, Carina Metzner, Dorota Religa, Maria Eriksdotter

**Affiliations:** 1 Division of Clinical Geriatrics, Department of Neurobiology, Care Sciences and Society, Karolinska Institutet, NEO, Blickagången 16, Huddinge, 141 52 Stockholm, Sweden; 2 Theme Aging, Karolinska University Hospital, Stockholm, Sweden; 3 Department of Health, Medicine and Caring Sciences, Linköping University, Linköping, Sweden; 4 Department of Clinical Science, Intervention and Technology (CLINTEC), Karolinska Institutet, Stockholm, Sweden; 5 Department of Renal Medicine, Karolinska University Hospital, Stockholm, Sweden; 6 Clinical Nutrition and Metabolism, Department of Public Health and Caring Sciences, Uppsala University, Uppsala, Sweden; 7 Department of Geriatrics, Capio Geriatrik Nacka AB, Nacka, Sweden

**Keywords:** COVID-19, Acute kidney injury, Older adults, In-hospital death

## Abstract

**Background:**

Research regarding COVID-19 and acute kidney injury (AKI) in older adults is scarce. We evaluated risk factors and outcomes of AKI in hospitalized older adults with and without COVID-19.

**Methods:**

Observational study of patients admitted to two geriatric clinics in Stockholm from March 1st to June 15th, 2020. The difference in incidence, risk factors and adverse outcomes for AKI between patients with or without COVID-19 were examined. Odds ratios (OR) for the risk of AKI and in-hospital death were obtained from logistic regression.

**Results:**

Three hundred-sixteen older patients were hospitalized for COVID-19 and 876 patients for non-COVID-19 diagnoses. AKI occurred in 92 (29%) patients with COVID-19 vs. 159 (18%) without COVID-19. The odds for developing AKI were higher in patients with COVID-19 (adjusted OR, 1.70; 95% confidence interval [CI] 1.04–2.76), low baseline kidney function as depicted by estimated glomerular filtration rate (eGFR) [4.19 (2.48–7.05), for eGFR 30 to  < 60 mL/min, and 20.3 (9.95–41.3) for eGFR < 30 mL/min], and higher C reactive protein (CRP) (OR 1.81 (1.11–2.95) in patients with initial CRP > 10 mg/L). Compared to patients without COVID-19 and without AKI, the risk of in-hospital death was highest in patients with COVID-19 and AKI [OR 80.3, 95% CI (27.3–235.6)], followed by COVID-19 without AKI [16.3 (6.28–42.4)], and by patients without COVID-19 and with AKI [10.2 (3.66–28.2)].

**Conclusions:**

Geriatric patients hospitalized with COVID-19 had a higher incidence of AKI compared to patients hospitalized for other diagnoses. COVID-19 and reduced baseline kidney function were risk factors for developing AKI. AKI and COVID-19 were associated with in-hospital death.

**Supplementary Information:**

The online version contains supplementary material available at 10.1007/s40620-021-01022-0.

## Introduction

Since December 2019, COVID-19 has become a global pandemic. Acute kidney injury (AKI) is common among COVID-19 patients [1–5], due to multifactorial COVID-19-related factors [6–8]. Previous studies from China, the United States and Europe showed that the incidence of AKI varied considerably from 1 to 80% among patients hospitalized with COVID-19 [1–5], and was especially high in intensive care units (ICUs) [4, 9]. AKI has also been considered a marker of severity of COVID-19 as well as a risk factor for COVID-19-related in-hospital death [10–12].

To date, there are few reports on the risk factors for AKI and post-AKI outcomes in COVID-19 in geriatric patients [13–16] and comparisons of AKI in geriatric patients with and without COVID-19 are also lacking. It is unknown whether geriatric patients with COVID-19 have similar risk factors for AKI and AKI-related adverse outcomes compared to their non-COVID-19 counterparts. What is well known, however, is that the risk of more severe illness and mortality from COVID-19 is higher in older patients [17–20].

On January 31st, 2020, Sweden had its first COVID-19 case and currently over 500,000 cases have been reported. The Stockholm region has been severely affected during the COVID-19 pandemic, and has suffered a high mortality rate in older patients [21]. The purpose of this study was to describe the incidence, risk factors, and outcomes for AKI in patients hospitalized with COVID-19 at two large geriatric clinics in Stockholm, and to determine whether there are risk factors for AKI unique to COVID-19 infection by comparison with patients treated for non-COVID-19 diagnoses in these clinics during the same period.

## Methods

### Study population

In the present study, we included all hospitalizations for patients who were admitted to two geriatric clinics in two hospitals in Stockholm, Sweden, from March 1st to June 15th, 2020. We excluded patients if (1) hospitalization lasted < 24 h, (2) they were undergoing renal replacement therapy (maintenance dialysis or renal transplant), or (3) serum creatinine measurements after admission were missing (Supplemental Fig. S1). A total of 1191 hospitalizations were eligible, comprising 316 patients hospitalized for COVID-19 and 875 hospitalized for non-COVID-19 diagnoses during the same period.

### COVID-19 diagnosis and covariates

The COVID-19 diagnosis was based on either a positive reverse transcriptase polymerase chain reaction (RT-PCR) analysis from nasopharyngeal swabs or, for patients with symptoms but with a negative RT-PCR, on a clinical diagnosis and a typical thoracic CT scan. Information on patient demographics, initial vital signs, laboratory data during hospitalization, medication and in-hospital death were collected through the hospital’s electronic health records. The definition of comorbidities was based on the International Classification of Diseases (ICD)-10 code that was obtained from discharge records [22]. Diabetes and dementia were further enriched with information on current prescription of related medications. These data were then used to create a Charlson Comorbidity Index (CCI) Score [23]. Since comorbidities were registered at the end of hospitalization they were only for description, and were not used to analyse to avoid reverse causation. The baseline kidney function as depicted by estimated glomerular filtration rate (eGFR) [24], was calculated from the lowest creatinine value measured during the hospital stay [25]. This is consistent with previous studies [14, 26] to baseline estimate kidney function, considering that we did not have kidney function data before hospitalization. We used eGFR < 30, 30–59 and 60** + **mL/min/1.73m^2^ categories to define the different stages of baseline kidney function, and the 60** + **mL/min/1.73m^2^ was used as the reference group [24].

### AKI and In-hospital death

AKI was defined as a 0.3 mg/dL (26.5 mmol/L) increase or a 50% increase in serum creatinine from baseline to the maximum values of in-hospital creatinine measurements for each patient [27]. Since creatinine values prior to hospitalization were not available in this study, we chose the minimum creatinine value during hospitalization as the baseline creatinine, as proposed by Siew et al [25]. The severity of AKI was defined as AKI stage 1–3 according to the Kidney Disease Improving Global Outcomes criteria [27]: stage (1) increase in creatinine by 0.3 mg/dL (26.5 mmol/L) or a 50–99% increase in creatinine; stage (2) 100–199% increase in creatinine; stage (3) 200% or more increase in creatinine or increase in creatinine to ≥ 4.0 mg/dL (353.6 mmol/L) or initiation of renal replacement therapy (Supplemental Table S1).

The secondary outcomes included (a) in-hospital death; (b) renal recovery of AKI in survivors at discharge. Renal recovery from AKI was defined as ≤ 50% increase in creatinine from baseline value to discharge according to the Acute Dialysis Quality Initiative (ADQI) [28]. Patients were censored at discharge from hospital, death, or transfer to other departments/clinics or other hospitals, whichever occurred first.

### Statistical analyses

Continuous variables were reported as mean ± standard deviation (SD) or median and interquartile range (IQR), and categorical variables were reported as a percentage. Chi-square and Wilcoxon rank sum test were used to test the differences between patients with and without AKI during hospitalization.

Logistic regression was used to test the association of covariates of interest (including diagnosis of COVID-19, admission sources, admitting hospital, age, sex, vital signs, lab values, baseline eGFR and medications) with the risk of incidence of AKI. To evaluate whether the same risks of AKI applied to patients with or without COVID-19, we performed subgroup analyses by the COVID-19 diagnosis. Baseline eGFR was also introduced as a cubic spline with the risk of incidence of AKI.

Logistic regression models were also used to estimate the risk of in-hospital death for patients with AKI compared to patients without AKI. Statistical interaction to evaluate whether COVID-19 modified the association between AKI and in-hospital death was modeled by including a product of COVID-19 and AKI in the regression model. We investigated associations between increase in percent (%) in serum creatinine and risk of in-hospital death using cubic splines, with knots at the 10th, 50th, and 90th percentile.

Data on CRP values, initial temperature, systolic blood pressure (SBP), diastolic blood pressure (DBP), pulse rate, and oxygen saturation (SpO_2_) were missing for 30(2.5%), 1(0.1%), 6(0.5%), 6 (0.5%), 3(0.3%), and 176(14.8%) patients, respectively. Missing data were handled with the use of multiple imputation with chained equations. All analyses were performed using R (https://www.r-project.org) and Stata version 16.0 (StataCorp, College Station, TX, USA).

### Ethical statement

The Swedish Ethical Review Authority approved the study (Dnr 2020-02146, and 2020-03345).

## Results

### Characteristics of patients with AKI and COVID-19 diagnosis

A total of 316 COVID-19 and 875 non-COVID-19 older adults were included. In the overall cohort of both groups, the mean age was 83 ± 9 years, 57% were women, baseline eGFR was 62 ± 23 mL/min, and the median number of creatinine measurements per patient during hospitalization was 2(IQR, 1–4). The most common comorbidity was hypertension (40%), followed by diabetes (37%), and dementia (30%). As compared to patients without COVID-19, patients with COVID-19 had a higher prevalence of hypertension (50% vs 36%), diabetes (46% vs 33%), and chronic obstructive pulmonary disease (COPD) (21% vs 14%), as well as higher CRP values at admission, higher initial temperature, lower DBP, lower SpO2 and more frequent prescription of low molecular weight heparin (LMWH)/non-vitamin-k oral anticoagulant (NOAC)/warfarin(84% vs 57%) and antibiotics(50% vs 41%) (Supplemental Table S2).

COVID-19 patients who developed AKI were more frequently diabetic (55% vs 42%), had lower baseline eGFR (57 ± 26 vs 67 ± 21 mL/min), increased initial median CRP value (78 vs 42 mg/L) and were more often on treatment with antibiotics at hospitalization(60% vs 46%) compared to the COVID-19 patients who did not develop AKI (Table 1). Non-COVID-19 AKI patients tended to be older (86 ± 8.4 vs 83 ± 8.6) and to have a higher prevalence of diabetes (41% vs 32%), chronic heart failure (32% vs 17%), lower baseline eGFR (47 ± 25 vs 64 ± 21 mL/min), increased initial CRP value( 53 vs 26 mg/L), and more prevalent use of diuretics (72% vs 55%), LMWH/NOAC/warfarin (64% vs 55%) and antibiotics (54% vs 38%) as compared to those without AKI (Table 1).

**Table 1:** Clinical characteristics of geriatric patients (n = 1191) according to the presence of acute kidney injury (AKI) and COVID-19 diagnosis, respectively

	COVID-19 (n = 316)			Other diagnosis (n = 875)		
	Non-AKI	with AKI	p-value	Non-AKI	with AKI	p value
Patients, n (%)	224 (71%)	92(29%)		716 (82%)	159(18%)	
Age, year mean ± SD	82.0 ± 8.6	82.8 ± 8.3	0.45	82.6 ± 8.6	85.5 ± 8.4	< 0.001
Women, n (%)	121 (54.0%)	43 (46.7%)	0.24	421 (58.8%)	88 (55.3%)	0.42
BMI, kg/m^2^ Mean ± SD (n = 675)	24.9 ± 6.2	24.3 ± 5.4	0.66	24.4 ± 5.3	24.9 ± 4.8	0.33
BMI < 18.5, n (%)	10 (12%)	4 (12%)	0.91	43 (9.3%)	7 (7.1%)	0.75
BMI 18.5 to < 25, n (%)	40 (49%)	19(56%)		237 (51.3%)	53 (54.1%)	
BMI 25 to < 30, n (%)	21 (26%)	8 (24%)		118 (25.5%)	22 (22.4%)	
BMI ≥ 30, n (%)	10 (12%)	3 (9%)		64 (13.9%)	16 (16.3%)	
Vital signs at admission, mean SD						
Temperature, °C (n = 1190)	37.1 ± 0.7	37.2 ± 0.9	0.25	37.0 ± 0.5	36.9 ± 0.7	0.42
SBP, mmHg (n = 1185)	132.7 ± 21.4	130.2 ± 20.7	0.34	132.2 ± 21.6	129.2 ± 22.4	0.11
DBP, mmHg (n = 1185)	71.5 ± 11.1	71.2 ± 13.2	0.88	73.5 ± 12.3	72.5 ± 13.4	0.37
Pulse rate, /min (n = 1188)	79.7 ± 15.5	82.5 ± 15.1	0.13	79.5 ± 14.1	79.6 ± 15.5	0.92
Saturation, % (n = 1015)	93.0 ± 5.9	92.5 ± 6.2	0.51	95.4 ± 4.8	94.8 ± 3.4	0.17
< 90%, n (%)	24(14.0%)	15(18.5%)	0.65	26 (4.2%)	10 (6.8%)	0.38
90 to < 94%, n (%)	54(31.6%)	25(30.9%)		120 (19.5%)	30 (20.4%)	
≥ 94%, n (%)	93(54.4%)	41(50.6%)		470 (76.3%)	107 (72.8%)	
Lab values						
eGFR, mL/min/1.73m^2^ baseline, mean ± SD	67.3 ± 21.1	56.9 ± 26.4	< 0.001	63.6 ± 20.6	46.5 ± 24.6	< 0.001
eGFR ≥ 60 mL/min, n (%)	146 (65.2%)	42 (45.7%)	< 0.001	429 (59.9%)	39 (24.5%)	< 0.001
eGFR 30–59 mL/min, n (%)	68 (30.4%)	34 (37.0%)		246 (34.4%)	77 (48.4%)	
eGFR < 30 mL/min, n (%)	10 (4.5%)	16 (17.4%)		41 (5.7%)	43 (27.0%)	
Minimum creatinine, mmol/L median (IQR)	66.0 (49.0, 89.5)	81.5 (57.5, 126.0)	< 0.001	70.0 (55.0, 89.0)	105.0 (72.0, 150.0)	< 0.001
Maximum creatinine, mmol/L median (IQR)	73.0 (56.5, 96.0)	130.0 (95.0, 216.5)	< 0.001	76.0 (61.0, 96.0)	159.0 (117.0, 220.0)	< 0.001
Number of creatinine measurements median (IQR)	2 (1,4)	6 (4,9)	< 0.001	2 (1,3)	5 (4,8)	< 0.001
CRP at admission, mg/L median (IQR) (n = 1160)	42.0 (19.0, 84.0)	77.5 (39.0, 117.5)	< 0.001	26.0 (9.0, 65.0)	53.0 (25.0, 120.0)	< 0.001
Comorbidities, n (%)						
CCI, median IQR	3.0 (1.0, 4.0)	3.0 (2.0, 5.0)	0.03	2.0 (0.0, 4.0)	3.0 (1.0, 5.0)	< 0.001
Hypertension	114 (50.9%)	44 (47.8%)	0.62	258 (36.0%)	60 (37.7%)	0.69
Diabetes	93 (41.5%)	51 (55.4%)	0.02	226 (31.6%)	65 (40.9%)	0.02
Chronic heart failure	49 (21.9%)	25 (27.2%)	0.31	124 (17.3%)	51 (32.1%)	< 0.001
Myocardial infarction	7 (3.1%)	6 (6.5%)	0.17	27 (3.8%)	5 (3.1%)	0.70
COPD	49 (21.9%)	18 (19.6%)	0.65	98 (13.7%)	20 (12.6%)	0.71
Asthma	12 (5.4%)	4 (4.3%)	0.71	21 (2.9%)	3 (1.9%)	0.47
Cancer	18 (8.0%)	7 (7.6%)	0.90	44 (6.1%)	12 (7.5%)	0.51
Stroke	19 (8.5%)	8 (8.7%)	0.95	42 (5.9%)	10 (6.3%)	0.84
Atrial fibrillation	64 (28.6%)	31 (33.7%)	0.37	173 (24.2%)	48 (30.2%)	0.11
MCI or dementia	55 (24.6%)	27 (29.3%)	0.38	225 (31.4%)	50 (31.4%)	1.00
Medication at admission, n (%)						
ACEIs	51 (22.8%)	25 (27.2%)	0.41	146 (20.4%)	41 (25.8%)	0.13
ARBs	55 (24.6%)	23 (25.0%)	0.93	169 (23.6%)	38 (23.9%)	0.94
Betablockers	120 (53.6%)	56 (60.9%)	0.24	381 (53.2%)	94 (59.1%)	0.18
CCBs	73 (32.6%)	32 (34.8%)	0.71	191 (26.7%)	55 (34.6%)	0.05
Diuretics	121 (54.0%)	56 (60.9%)	0.26	395 (55.2%)	115 (72.3%)	< 0.001
Statins	79 (35.3%)	38 (41.3%)	0.31	247 (34.5%)	69 (43.4%)	0.04
LMWH/NOAC/warfarin	189 (84.4%)	77 (83.7%)	0.88	393 (54.9%)	102 (64.2%)	0.03
Antiplatelets	77 (34.4%)	40 (43.5%)	0.13	215 (30.0%)	58 (36.5%)	0.11
NSAIDs	8 (3.6%)	2 (2.2%)	0.52	42 (5.9%)	5 (3.1%)	0.17
Glucocorticoids	46 (20.5%)	16 (17.4%)	0.52	104 (14.5%)	27 (17.0%)	0.43
Antibiotics	104 (46.4%)	55 (59.8%)	0.03	269 (37.6%)	86 (54.1%)	< 0.001
Outcomes						
AKI severity, n (%)						0.04
Stage 1		72 (78.3%)			142 (89.3%)	
Stage 2		15 (16.3%)			11 (6.9%)	
Stage 3		5 (5.4%)			6 (3.8%)	
In-hospital death, n (%)	29 (12.9%)	35 (38.0%)	< 0.001	7 (1.0%)	19 (11.9%)	< 0.001
Stay in hospital, days Mean SD	8.8 ± 6.0	13.2 ± 7.5	< 0.001	6.8 ± 3.8	12.0 ± 7.3	< 0.001

*SD* standard deviation, *IQR* interquartile range, *AKI* acute kidney injury, *SBP* systolic BP, *DBP* diastolic BP, *eGFR* estimated glomerular filtration rate, *CRP* C-reactive protein, *CCI* Charlson Comorbidity Index, *COPD* chronic obstructive pulmonary disease, *MCI* mild cognitive impairment, *ACEIs* angiotensin-converting enzyme inhibitors, *ARBs* angiotensin receptor blockers, *CCBs* calcium channel blockers, *LMWH* low molecular weight heparin, *NOAC* non-vitamin-k oral anticoagulant, *NSAIDs* nonsteroidal anti-inflammatory drugs

### Incidence and severity of AKI

AKI occurred in 92 (29%) patients with COVID-19 compared to 159 (18%) of those with other diagnoses who were hospitalized during the same pandemic period (p < 0.001). Among AKI patients with a COVID-19 diagnosis, 78% of AKI was stage 1, 16% stage 2, and 5.4% stage 3. Among patients with other diagnoses, 89% of AKI was stage 1, 7% stage 2, and 4% stage 3; none of the patients in AKI stage 3 required renal replacement therapy (i.e. dialysis). Severity of AKI differed significantly between patients with COVID-19 compared to patients with other diagnoses (p < 0.05) (Table 1).

### Risk factors for incidence of AKI during hospitalization

Overall, the odds of developing AKI were higher in patients with COVID-19 (OR, 1.70; 95% CI 1.04–2.76), in patients with reduced baseline kidney function [4.19 (2.48–7.05) when eGFR was 30–59 mL/min, and 20.3 (9.95–41.3) when eGFR was < 30 mL/min], and in patients with initial CRP > 10 mg/L [1.81 (1.11–2.95)] (Table 2).

**Table 2:** Odds ratios for incidence of AKI during hospitalization

	Whole cohort (n = 1191)		COVID-19 (n = 316)		Other diagnoses (n = 875)	
	Adjusted OR	95% CI	Adjusted OR	95% CI	Adjusted OR	95% CI
COVID-19	1.70*	1.04,2.76	–	–	–	–
Age categories						
Age < 80	Reference		Reference		Reference	
Age 80–90	1.28	0.75,2.17	3.57*	1.32,9.66	0.77	0.38,1.56
Age > 90	1.54	0.83,2.89	1.17	0.36,3.74	1.52	0.68,3.41
Women	0.73	0.47,1.12	0.53	0.24,1.21	0.91	0.52,1.60
Vital signs at admission						
Temperature, °C	1.01	0.74,1.37	1.06	0.64,1.75	0.89	0.57,1.38
SBP, mmHg	0.99	0.98,1.00	0.98	0.96,1.00	0.99	0.97,1.00
DBP, mmHg	1.01	0.99,1.03	1.01	0.98,1.05	1.01	0.98,1.03
Pulse rate, /min	1.01	1.00,1.03	1.01	0.98,1.04	1.01	0.99,1.03
Saturation, < 90%	1.45	0.71,2.98	1.98	0.70,5.59	2.18	0.70,6.76
Lab values						
Baseline kidney function						
eGFR ≥ 60 mL/min	Reference		Reference		Reference	
eGFR 30–59 mL/min	4.19***	2.48,7.05	2.94*	1.17,7.34	5.55***	2.74,11.2
eGFR < 30 mL/min	20.3***	9.95,41.3	9.93**	2.32,42.5	35.0***	14.0,87.7
Number of creatinine measurements	1.71***	1.56,1.86	1.55***	1.34,1.78	1.88***	1.66,2.13
CRP > 10 mg/L	1.81*	1.11,2.95	2.28	0.97,5.36	2.05*	1.07,3.95
Medication at admission						
ACEIs	1.14	0.69,1.90	1.21	0.44,3.32	1.17	0.61,2.26
ARBs	1.05	0.63,1.76	0.78	0.30,2.06	1.22	0.63,2.34
Betablockers	0.68	0.43,1.06	0.72	0.31,1.65	0.66	0.36,1.19
CCBs	1.24	0.78,1.96	1.04	0.45,2.39	1.57	0.85,2.90
Diuretics	1.07	0.66,1.73	1.08	0.46,2.56	1.14	0.60,2.18
Statins	1.44	0.91,2.28	1.62	0.68,3.85	1.57	0.86,2.87
LMWH/NOAC/warfarin	1.06	0.65,1.73	0.62	0.22,1.79	0.99	0.54,1.82
Antiplatelets	1.19	0.74,1.92	1.49	0.63,3.50	1.11	0.58,2.13
NSAIDs	1.58	0.60,4.14	1.12	0.13,10.1	2.07	0.65,6.67
Glucocorticoids	1.08	0.63,1.87	0.63	0.22,1.76	1.41	0.70,2.84
Antibiotics	1.12	0.73,1.72	1.63	0.74,3.59	0.93	0.52,1.63
Admission source						
From home	Reference		Reference		Reference	
From nursing home	1.39	0.40,4.90	0.66	0.04,10.4	2.10	0.45,9.86
From other clinic	1.10	0.71,1.72	0.93	0.39,2.21	1.39	0.79,2.46
Admission hospital						
Clinic 1 vs clinic 2	1.20	0.71,2.02	1.49	0.56,3.98	0.92	0.46,1.85

*OR* odds ratios, *CI* confidence interval, *AKI* acute kidney injury, *SBP* systolic BP, *DBP* diastolic BP, *eGFR* estimated glomerular filtration rate, *CRP* C-reactive protein, *ACEIs* angiotensin-converting enzyme inhibitors, *ARBs* angiotensin receptor blockers, *CCBs* calcium channel blockers, *LMWH* low molecular weight heparin, *NOAC* non-vitamin-k oral anticoagulant, *NSAIDs* nonsteroidal anti-inflammatory drugs. *p<0.05, **p<0.01, ***p<0.001

Subgroup analyses by COVID-19 diagnosis showed that reduced baseline kidney function increased the risk of AKI in patients with or without COVID-19 [2.94 (1.17–7.34) when eGFR was 30–59 mL/min and 9.93 (2.32–42.5) when eGFR was < 30 mL/min for COVID-19; 5.55 (2.74–11.2) when eGFR was 30–59 mL/min and 35.0 (14.0–87.7) when eGFR was < 30 mL/min for non-COVID-19 and other diagnoses, respectively]. Age 80–90 years was associated with higher odds of AKI in patients with COVID-19 [3.57(1.32–9.66)]. Increased initial CRP value (> 10 mg/L) was associated with higher odds of AKI [2.05 (1.07–3.95)] in patients with other diagnoses but did not become significant in patients with COVID-19 [2.28 (0.97–5.36)] (Table 2). When modeling eGFR as a continuous metric using splines, we observed an increased OR when eGFR was  less than 60 mL/min in both COVID-19 and with other diagnoses (Supplemental Fig. S2).

### AKI and in-hospital death

In patients with COVID-19, in-hospital death was 38% among those with AKI compared to 13% in those without AKI. By comparison, in patients without COVID-19, in-hospital death was 12% in those with AKI compared to 1% in those without AKI. In multivariable logistic regression, AKI was associated with a higher risk of in-hospital death in patients with COVID-19 [OR 8.64 (95% CI 3.25–23.0)]. Similar associations were also found in patients with other diagnoses [10.2 (2.89–35.9)]. No statistically significant interaction terms were observed for the product of COVID-19 and AKI with in-hospital death (P_interaction_ = 0.22) (Table 3). In multivariable adjusted analyses, a higher change in serum creatinine was associated with a higher risk of in-hospital death, with statistically significant associations for changes in serum creatinine > 50% (Supplemental Fig. S3). We observed an increased risk of in-hospital death across the severity of AKI stages and significant differences among AKI stages (p < 0.05) (Supplemental Table S3).

**Table 3:** Odds ratios and 95% CIs for in-hospital death by the presence of acute kidney injury and COVID-19 in geriatric patients

	Whole cohort (n = 1191)		COVID-19 (n = 316)		Other diagnoses (n = 875)	
	Adjusted OR	95% CI	Adjusted OR	95% CI	Adjusted OR	95% CI
Non-COVID-19 non-AKI	Reference				Reference	
Non-COVID-19 and AKI	10.2***	3.66,28.2			10.2***	2.89,35.9
COVID-19 and non-AKI	16.3***	6.28,42.4	Reference			
COVID-19 and AKI	80.3***	27.3,235.6	8.64***	3.25,23.0		
Age categories						
Age < 80	Reference		Reference		Reference	
Age 80–90	1.75	0.84,3.61	1.04	0.38,2.85	3.59	0.80,16.0
Age > 90	2.26	1.00,5.13	1.65	0.54,5.06	2.84	0.58,14.0
Women	0.67	0.38,1.18	0.57	0.26,1.28	0.85	0.30,2.42
Vital signs at admission						
Temperature, ^o^C	1.10	0.76,1.60	1.22	0.74,2.02	0.76	0.35,1.66
SBP, mmHg	0.99	0.98,1.01	1.00	0.98,1.02	1.00	0.97,1.03
DBP, mmHg	1.00	0.98,1.03	1.00	0.97,1.04	0.99	0.94,1.04
Pulse rate, /min	1.01	0.99,1.03	1.00	0.98,1.03	1.03	1.00,1.07
Saturation categories						
< 90%	5.84***	2.63,13.0	10.5***	3.31,33.3	6.67*	1.42,31.3
90 to < 94%	1.36	0.69,2.66	2.14	0.84,5.44	0.99	0.26,3.73
≥ 94%	Reference		Reference		Reference	
Lab values						
Baseline kidney function						
eGFR ≥ 60 mL/min	Reference		Reference		Reference	
eGFR 30–59 mL/min	1.52	0.77,3.00	1.93	0.77,4.85	1.46	0.37,5.81
eGFR < 30 mL/min	5.01***	2.12,11.8	9.74**	2.44,38.9	6.43*	1.49,27.8
Number of creatinine measurements	0.90*	0.81,1.00	0.82*	0.70,0.95	1.11	0.94,1.30
CRP > 10 mg/L	1.15	0.62,2.14	1.73	0.75,3.99	0.45	0.12,1.70
Medication at admission						
ACEIs	0.75	0.39,1.47	0.47	0.18,1.22	1.63	0.52,5.09
ARBs	0.73	0.36,1.47	0.68	0.25,1.84	1.01	0.29,3.51
Betablockers	0.78	0.43,1.41	0.35*	0.15,0.84	2.92	0.93,9.15
CCBs	0.80	0.43,1.48	1.36	0.61,3.02	0.10**	0.02,0.51
Diuretics	1.25	0.68,2.31	1.42	0.63,3.18	0.65	0.21,2.04
Statins	0.42**	0.22,0.81	0.48	0.20,1.16	0.47	0.14,1.50
LMWH/NOAC/warfarin	1.99	0.95,4.17	2.86	0.89,9.19	1.46	0.44,4.86
Antiplatelets	2.38**	1.29,4.37	1.94	0.86,4.40	3.44*	1.06,11.2
NSAIDs	1.16	0.29,4.68	1.07	0.13,8.82	1.19	0.10,14.2
Glucocorticoids	1.63	0.82,3.26	1.64	0.65,4.15	2.11	0.57,7.74
Antibiotics	1.39	0.78,2.48	1.36	0.61,3.04	1.30	0.45,3.81
Admission source						
From home	Reference		Reference		Reference	
From nursing home	0.39	0.04,3.37	0.60	0.05,7.85	–	–
From other clinic	0.68	0.37,1.25	0.44	0.18,1.09	1.42	0.49,4.18
Admission hospital						
Clinic 1 vs clinic 2	3.68**	1.63,8.29	4.67**	1.56,14.0	2.90	0.47,17.8

*OR* odds ratios, *CI* confidence interval, *AKI* acute kidney injury, *SBP* systolic BP, *DBP* diastolic BP, *eGFR* estimated glomerular filtration rate, *CRP* C-reactive protein, *ACEIs* angiotensin-converting enzyme inhibitors, *ARBs* angiotensin receptor blockers, *CCBs* calcium channel blockers, *LMWH* low molecular weight heparin, *NOAC* non-vitamin-k oral anticoagulant, *NSAIDs* nonsteroidal anti-inflammatory drugs. *p<0.05, **p<0.01, ***p<0.001

In the whole cohort of 1,191 patients, the risk of in-hospital death was highest among patients with COVID-19 and with AKI [80.3 (27.3–235.6)], followed by patients with COVID-19 without AKI [16.3 (6.28–42.4)], and finally by non-COVID-19 patients with other diagnosis and AKI [10.2 (3.66–28.2)]. Where patients with other diagnoses and non-AKI was used as the reference group (Table 3). Similar results were observed when we excluded 63 patients from our sensitivity analyses who were transferred to other departments or hospitals (Supplemental Table S4).

### Recovery from AKI at discharge

We did not find any significant association between COVID-19 infection and recovery from AKI at discharge. In addition, the severity of the AKI stages was not associated with recovery from AKI in patients with COVID-19 or in those with other diagnoses (Table 4).

**Table 4:** Recovery from AKI at discharge

	COVID-19 (n = 57)		Other diagnoses (n = 140)		p value
	Recovery yes	Recovery no	Recovery yes	Recovery no	
AKI at any-stage					0.64
	50 (88%)	7 (12%)	126 (90%)	14 (10%)	
AKI severity					
Stage 1	42 (89%)	5 (11%)	115 (91%)	12 (9%)	
Stage 2	8 (80%)	2 (20%)	7 (78%)	2 (22%)	
Stage 3	–	–	4 (100%)	0	
P trend among AKI stages	0.41		0.37		

*AKI* acute kidney injury

## Discussion

In this Swedish cohort of geriatric patients hospitalized with or without COVID-19, older adults with COVID-19 had a higher incidence of AKI compared to those without COVID-19 who were hospitalized with other diagnoses. COVID-19 infection and poor baseline kidney function are risk factors for AKI during hospitalization. Furthermore, we demonstrated that AKI was associated with higher in-hospital death in both COVID-19 and non-COVID-19 patients.

In our cohort, 29% of older adults with COVID-19 developed AKI during hospitalization, which is a higher proportion than was published for cohorts from China [13, 16], less pronounced compared to patients from the USA [14, 15] and from intensive care units [4, 9]. In one study from China that collectively included 701 COVID-19 cases with a median age of 63 years, the incidence of AKI was only 5.1% [16]. Another Chinese study showed that in 882 older COVID-19 patients with a median age of 71 years, consisting of 50% men, 13% developed AKI [13]. Our 29% incidence of AKI is lower than what has been reported in studies from the USA. In two studies involving 14 hospitals in New York that included 5,800 individuals, median age 64–71 years, 56–61% males, admitted with COVID-19, AKI occurred in 37% and 55%, respectively [14, 15]. In addition, a recent meta-analysis among patients admitted to the ICU showed that the pooled AKI events were 29.2% (4330 patients from 23 studies) [4]. The discrepancy between these reports and our study might be explained by differences in patient population, geographic location and guidelines regarding hospital admission. In our study, the proportion of COVID-19 patients with AKI stage 2 or 3 was higher compared to patients with other diagnoses. In this population none of the patients in AKI stage 3 received renal replacement therapy ([RRT], e.g., dialysis). This may have been due to either transient AKI which improved before the patients met indications for dialysis or, more likely, to the fact that the treating physicians chose not to send older patients to dialysis because it was deemed futile in view of their serious general conditions. Whether these differences in AKI incidence among studies reflect differences in severity of COVID-19 is not clear. In the early spring in Stockholm, with exponentially increasing numbers of COVID-19 patients needing hospital care, the geriatric clinics played an important role in treating, administering and upholding care guidelines for geriatric patients with COVID-19 and many severe patients were admitted to geriatrics units. Another explanation could be that the different age among studies. Patients admitted for COVID-19 in our study and in US studies tended to be older compared to patients from China [29]. In addition, the pattern of comorbidities differed in our geriatric population compared to populations elsewhere; the 40% rate of hypertension, 37% of diabetes, and 16% of COPD in our cohort were much higher than in cohorts from China (33%, 14% and 1.9% respectively) [16]. This could suggest that management of comorbidities will play a large role in preventing AKI during hospitalization.

We found that COVID-19 infection and reduced baseline kidney function may be risk factors for the development of AKI. Several plausible mechanisms have been proposed to explain the link between COVID-19 and AKI. First, the kidney may be a target organ in COVID-19 because SARS-CoV-2 directly damage the kidney via the angiotensin-converting enzyme 2 (ACE2) pathway [6–8]. Studies of biopsy samples showed that the presence of SARS-CoV-2 particles in proximal tubule cells and podocytes may support this hypothesis [30, 31]. Second, complications including immune response dysregulation, hypercoagulability, acute tubular necrosis by dehydration, sepsis and hemodynamic instability in the course of COVID-19 infections outside the kidney are associated with AKI [32]. We report an association between the reduced baseline kidney function (assessed by eGFR) and the risk of developing AKI. However, our in-hospital “baseline” kidney function evaluation may have underestimated the risk of kidney function on AKI, and moreover, in our study it was not known whether renal dysfunction was caused by AKI or by a pre-existing chronic renal disease. Our results show similar magnitudes and are consistent with previous studies which also used the lowest in-hospital value of creatinine to calculate eGFR as baseline kidney function [14, 26]. However, the baseline kidney function in patients with AKI in our study was higher than in studies using admission values to estimate baseline kidney function [15, 16]. In addition, it is not yet known whether COVID-19 will increase the risk of CKD long-term in different populations. However in a study from New York among survivors with AKI needing dialysis, 30.6% remained on dialysis at discharge [33].

Our principal finding was that AKI in COVID-19 patients was associated with an eightfold higher in-hospital mortality rate compared to those who did not develop AKI in COVID-19. Moreover, the mortality risk after adjusting for age, gender, lab values, initial vital signs and medications was 80 times higher for those with COVID-19 and AKI, and 10 times higher for those withoutCOVID-19 and with AKI compared to patients without COVID-19 and without AKI. This result showed the same direction but larger magnitude than in several previous meta-analyses showing increased in-hospital mortality in patients with COVID-19 who developed AKI (pooled risk ratios 5–15) [4, 10–12]. However, these pooled risk ratios were based on the crude numbers, and our estimates were adjusted for many confounders and compared to non-COVID-19 and non-AKI. Our findings are also similar to other severe infectious diseases associated with AKI. One meta-analysis that collectively included 226 studies from 50 countries of critically ill patients with Influenza A(H1N1) reported a 36% incidence of AKI and 17–44% increased mortality [34]. A recent meta-analysis comparing different coronavirus infections showed that the incidence of AKI in severe acute respiratory syndrome (SARS) infection was only 6–16%, while AKI in patients with Middle East respiratory syndrome (MERS) coronavirus infection was as high as 27–49%. On the other hand, mortality after developing AKI was 80–90% in SARS and 60–70% in MERS [35]. In general, AKI mortality in COVID-19 in our study is higher than in H1N1 infection, but lower than in SARS or MERS.

Strengths and limitations must be considered when interpreting the results of the present study. The main strength was the inclusion of a relatively large sample of hospitalized older patients including both COVID-19 and non-COVID-19 patients, with a wealth of information concerning potential risk factors and confounders, as well as access to health records during the hospital stay. One important limitation of the study is that we had no information on baseline creatinine measurement prior to hospitalization. Instead, we used the lowest in-hospital creatinine value as the baseline creatinine in the analysis as a proxy for pre-hospital creatinine. We acknowledge that relying on the lowest in-hospital creatinine value may have led to under-estimation of the AKI events and, consequently, to overestimation of AKI-associated events, nonetheless the use of the lowest in-hospital creatinine has been proven to be appropriate and is widely used in many studies [14, 25, 26]. The lack of information on pre-hospital kidney function or urine tests is another limitation of the study. This makes it difficult to study COVID-19 in the special population with chronic kidney disease. In addition, information on the time of onset of peak creatinine was not available, but in clinical practice, treating hospitalized COVID-19 patients follow up of kidney function including repeated creatinine analyses is recommended within 1–7 days (Swedish clinical guidelines published by the Swedish Infectious disease association https://www.internetmedicin.se/behandlingsoversikter/infektion/covid-19/). We acknowledge that the lack of RRT in AKI stage 3 may add a confounding bias in the association of AKI and mortality. Another limitation is that we do not have data on long-term mortality and therefore have reported only in-hospital deaths. The mortality rate may therefore be underestimated. COVID-19 in geriatric patients with a longer follow-up is planned in future studies. Finally, as in all observational and register-based analyses, we acknowledge the possibility of residual and unknown confounders such as socio-economic status and body mass index.

## Conclusion

In summary, our study shows that in this Swedish cohort of geriatric patients, those with COVID-19 had a higher incidence of AKI compared to non-COVID-19 patients. COVID-19 and lower baseline kidney function may be a risk factor for AKI. In-hospital mortality risk was highest in patients with AKI and COVID-19. Since the development of AKI is one of the most important risk factors for mortality in COVID-19 patients, focus on, and optimal management of, AKI may improve the outcome of COVID-19 in geriatric patients.

## Supplementary Information

Below is the link to the electronic supplementary material.

Supplementary file1 (DOCX 291 KB)
