# Supplementary material for: Acute kidney injury and mortality risk in older adults with COVID-19
Source: J Nephrol. 2021 Mar 22;34(2):295–304. doi: 10.1007/s40620-021-01022-0 (PMC7982881; doi:10.1007/s40620-021-01022-0)

**Supplemental Table S1:** A) Definitions of AKI according to Kidney Disease Improving Global Outcomes criteria; B) Definitions of recovery from AKI

1. Definitions of AKI according to Kidney Disease Improving Global Outcomes criteria

|  | **Definitions** |
| --- | --- |
| AKI | Increase in creatinine > 0.3 mg/dl (26.5 mmol/L) within 48 hours or  Creatinine increase in % > 50% within 7 days |
| **AKI Stage** |  |
| Stage 1 | Increase in creatinine > 0.3 mg/dl (26.5 mmol/L) or  Creatinine increase in % > 50% and ≤ 100% |
| Stage 2 | Creatinine increase in % > 100% and ≤ 200% |
| Stage 3 | Creatinine increase in % > 200% or  Increase in creatinine > 4 mg/dl (353.6 mmol/L) or  Initiating renal replacement therapy |

1. Definitions of recovery from AKI according to Acute Dialysis Quality Initiative

|  | **Definitions** |
| --- | --- |
| Recovery from AKI | creatinine change in % ≤ 50% at discharge |

Abbreviations: AKI, acute kidney injury**.**

**Supplemental Table S2.** Characteristics of patients with COVID-19 diagnosis and patients with non-COVID-19 and hospitalized for other diagnoses

|  | **Whole cohort** | **COVID-19** | **Other diagnosis** | **p-value** |
| --- | --- | --- | --- | --- |
| **Patients, n (%)** | **1,191** | **316** | **875** |  |
| **Age, year mean**±**SD** | **82.9± 8.6** | **82.2± 8.5** | **83.1± 8.6** | **0.11** |
| **Women, n (%)** | **673 (56.5%)** | **164 (51.9%)** | **509 (58.2%)** | **0.05** |
| **BMI, kg/m^2^**  **mean±SD (n=675)** | **24.5± 5.3** | 24.5**±** 5.2 | 24.7 **±** 6.0 | 0.67 |
| **BMI <18.5, n (%)** | 64 (9.5%) | 50 (8.9%) | 14 (12.2%) | **0.64** |
| **BMI 18.5-<25, n (%)** | 349 (51.7%) | 290 (51.8%) | 59 (51.3%) |  |
| **BMI 25-<30,** **n (%)** | 169 (25.0%) | 140 (25.0%) | 29 (25.2%) |  |
| **BMI ≥30,** **n (%)** | 93 (13.8%) | 80 (14.3%) | 13 (11.3%) |  |
| **Vital signs at admission, mean SD** |  |  |  |  |
| **Temperature, ^o^C (n=1190)** | **37.0 ± 0.6** | **37.1± 0.8** | **37.0± 0.6** | **<0.001** |
| **SBP, mmHg (n=1185)** | **131.8± 21.6** | **132.0± 21.2** | **131.7± 21.8** | **0.82** |
| **DBP, mmHg (n=1185)** | **72.8± 12.3** | **71.4± 11.7** | **73.3 ± 12.5** | **0.02** |
| **Pulse rate, /min (n=1188)** | **79.8± 14.6** | **80.5± 15.4** | **79.5± 14.3** | **0.30** |
| **Saturation, % (n=1015)** | **94.7± 5.1** | **92.8± 5.9** | **95.3 ± 4.6** | **<0.001** |
| **<90%, n (%)** | 75 (7.4%) | 39 (15.5%) | 36 (4.7%) | **<0.001** |
| **90%-<94%** | 229 (22.6%) | 79 (31.3%) | 150 (19.7%) |  |
| **≥94%** | 711 (70.0%) | 134 (53.2%) | 577 (75.6%) |  |
| **Lab values** |  |  |  |  |
| **eGFR, ml/min/1.73m^2^ baseline, mean**±**SD** | **61.5±22.6** | **64.3±23.2** | **60.5±22.4** | **0.01** |
| **Minimum creatinine, mmol/L median (IQR)** | **72.0 (55.0, 99.0)** | **69.0 (51.0, 96.0)** | **73.0 (56.0, 99.0)** | **0.05** |
| **Maximum creatinine, mmol/L median (IQR)** | **82.0 (62.0, 119.0)** | **83.0 (61.0, 124.5)** | **82.0 (63.0, 117.0)** | **0.76** |
| **Number of creatinine measurements median (IQR)** | **2 (1,4)** | **3(2,6)** | **2(1,4)** | **<0.001** |
| **CRP at admission, mg/L median (IQR) (n=1,160)** | **37.0 (13.0, 82.0)** | **50.0 (24.0, 101.0)** | **30.0 (11.0, 75.0)** | **<0.001** |
| **Comorbidities, n (%)** |  |  |  |  |
| **CCI, median IQR** | **2.0 (1.0, 4.0)** | **3.0 (1.0, 4.0)** | **2.0 (1.0, 4.0)** | **<0.001** |
| **Hypertension** | 476 (40.0%) | 158 (50.0%) | 318 (36.3%) | <0.001 |
| **Diabetes** | **435 (36.5%)** | **144 (45.6%)** | **291 (33.3%)** | **<0.001** |
| **Chronic heart failure** | **249 (20.9%)** | **74 (23.4%)** | **175 (20.0%)** | **0.20** |
| **Myocardial Infarction** | **45 (3.8%)** | **13 (4.1%)** | **32 (3.7%)** | **0.72** |
| **COPD** | **185 (15.5%)** | **67 (21.2%)** | **118 (13.5%)** | **0.001** |
| **Asthma** | **40 (3.4%)** | **16 (5.1%)** | **24 (2.7%)** | **0.05** |
| **Cancer** | **81 (6.8%)** | **25 (7.9%)** | **56 (6.4%)** | **0.36** |
| **Stroke** | **79 (6.6%)** | **27 (8.5%)** | **52 (5.9%)** | **0.11** |
| **Atrial fibrillation** | **316 (26.5%)** | **95 (30.1%)** | **221 (25.3%)** | **0.10** |
| **MCI or Dementia** | **357 (30.0%)** | **82 (25.9%)** | **275 (31.4%)** | **0.07** |
| **Medication at admission,**  **n (%)** |  |  |  |  |
| **ACEIs** | **263 (22.1%)** | **76 (24.1%)** | **187 (21.4%)** | **0.33** |
| **ARBs** | **285 (23.9%)** | **78 (24.7%)** | **207 (23.7%)** | **0.71** |
| **Betablockers** | **651 (54.7%)** | **176 (55.7%)** | **475 (54.3%)** | **0.67** |
| **CCBs** | **351 (29.5%)** | **105 (33.2%)** | **246 (28.1%)** | **0.09** |
| **Diuretics** | **687 (57.7%)** | **177 (56.0%)** | **510 (58.3%)** | **0.48** |
| **Statins** | **433 (36.4%)** | **117 (37.0%)** | **316 (36.1%)** | **0.77** |
| **LMWH/NOAC/warfarin** | **761 (63.9%)** | **266 (84.2%)** | **495 (56.6%)** | **<0.001** |
| **Antiplatelets** | **390 (32.7%)** | **117 (37.0%)** | **273 (31.2%)** | **0.06** |
| **NSAIDs** | **57 (4.8%)** | **10 (3.2%)** | **47 (5.4%)** | **0.12** |
| **Glucocorticoids** | **193 (16.2%)** | **62 (19.6%)** | **131 (15.0%)** | **0.06** |
| **Antibiotics** | **514 (43.2%)** | **159 (50.3%)** | **355 (40.6%)** | **0.003** |
| **Outcomes** |  |  |  |  |
| **Stay in hospital days,**  **mean± SD** | **8.4 ± 5.6** | **10.1± 6.7** | **7.8± 5.1** | **<0.001** |
| **In-hospital death, %** | **90 (7.6%)** | **64 (20.3%)** | **26 (3.0%)** | **<0.001** |

Abbreviations: SD, standard deviation; IQR, interquartile range; AKI, acute kidney injury; SBP, systolic BP; DBP, diastolic BP; eGFR, estimated glomerular filtration rate; CRP, C-reactive protein; CCI, Charlson Comorbidity Index; COPD, chronic obstructive pulmonary disease; MCI, mild cognitive impairment; ACEIs, angiotensin-converting enzyme inhibitors; ARBs, angiotensin receptor blockers; CCBs, calcium channel blockers; LMWH, low molecular weight heparin; NOAC, non-vitamin-k oral anticoagulant; NSAIDs, nonsteroidal anti-inflammatory drugs.

**Supplemental Table S3.** Odds ratios and 95% CIs for in-hospital death by the severity of acute kidney injury and COVID-19 in geriatric patients

|  | **Death, n (%)** | **OR** | **95%CI** |  |
| --- | --- | --- | --- | --- |
| **Covid-19 patients (n=316)** |  |  |  |  |
| **Non-AKI (n=224)** | 29 (13%) | Reference |  |  |
| **AKI stage 1(n=72)** | 25 (35%) | 7.95*** | 2.81,22.5 |  |
| **AKI stage 2(n=15)** | 5 (33%) | 15.0** | 2.29,97.9 |  |
| **AKI stage 3(n=5)** | 5 (100%) | - | - |  |
| **P for trend** |  | <0.001 |  |  |
| **Other diagnoses (n=875)** |  |  |  |  |
| **Non-AKI (n=716)** | 7 (1%) | Reference |  |  |
| **AKI stage 1(n=142)** | 15 (11%) | 10.1*** | 2.76,37.1 |  |
| **AKI stage 2(n=11)** | 2 (18%) | 12.0* | 1.02,141.1 |  |
| **AKI stage 3(n=6)** | 2 (33%) | 441.3*** | 18.4,10573.3 |  |
| **P for trend** |  | <0.001 |  |  |

Model adjusted for age (categories) and sex, initial vital signs (temperature, SBP, DBP, pulse rate, and saturation categories), CRP value (>10mg/L), number of Cr measurements, baseline eGFR (categories) and medication (ACEIs, ARBs, beta-blockers, CCBs, diuretics, statins, LMWH/NOAC/warfarin, antiplatelet, NSAIDS, glucocorticoids and antibiotics), admission sources, and admission hospital.

Abbreviations: OR, odds ratio: CI, confidence interval; AKI, acute kidney injury

**Supplemental Table S4.** Odds ratios and 95% CIs for in-hospital death by the presence of acute kidney injury and COVID-19 in geriatric patients by excluding 63 patients transferred to other departments or hospitals

|  | **Whole cohort (n=1,191)** | | **COVID-19 (n=316)** | | **Other diagnoses (n=875)** | |
| --- | --- | --- | --- | --- | --- | --- |
|  | **Adjusted OR** | **95% CI** | **Adjusted OR** | **95% CI** | **Adjusted OR** | **95% CI** |
| **Non-COVID-19 and non-AKI** | Reference |  |  |  | Reference |  |
| **Non-COVID-19 and AKI** | 10.6*** | 3.82,29.5 |  |  | 10.6*** | 3.01,37.0 |
| **COVID-19 and non-AKI** | 16.9*** | 6.46,44.5 | Reference |  |  |  |
| **COVID-19 and AKI** | 82.5*** | 27.8,244.8 | 9.10*** | 3.27,25.3 |  |  |

Model adjusted for age (categories) and sex, initial vital signs (temperature, SBP, DBP, pulse rate, and saturation categories), CRP value (>10mg/L), number of Cr measurements, baseline eGFR (categories) and medication (ACEIs, ARBs, beta-blockers, CCBs, diuretics, statins, LMWH/NOAC/warfarin, antiplatelet, NSAIDS, glucocorticoids and antibiotics), admission sources, and admission hospital.

Abbreviations: OR, odds ratio: CI, confidence interval; AKI, acute kidney injury

**Supplemental Figure S1 Flow chart of patient selection.**

Abbreviations: AKI, acute kidney injury

Hospitalizations at two geriatric clinics during the COVID-19 pandemic (**n=1,401)**

1. With <24h hospitalization: n=18

2. Patients on dialysis: n=8

3. Patients with kidney transplant: n=3

4. >1 creatinine value missing post-admission : n=181

Hospitalized due to other diagnoses **(n=875)**

**n=159 (18%)** with AKI

**n=716** without AKI

Hospitalized with confirmed **COVID-19 (n=316)**

**n=92 (29%)** with AKI

**n=224** without AKI

Enrolled in the study

**(n=1,191)**

**Supplemental Figure S2.** Odds ratios for risk of AKI events by baseline eGFR (ml/min increase, continuous variable) using cubic splines, in patients with (A) and without (B) COVID-19. Model adjusted for age, sex, initial vital signs (temperature, SBP, DBP, pulse rate, and saturation), CRP value, number of Cr measurements, baseline eGFR value (continuous), medication (ACEIs, ARBs, beta-blockers, CCBs, diuretics, statins, LWMH/NOAC/warfarin, anti-platelets, NSAIDS, glucocorticoids and antibiotics), admission sources, and admission hospital. Data were reported as Odds ratios (OR) and 95% confidence intervals (CI). In the lower part, percentage of participants across baseline eGFR in older adults hospitalized with and without COVID-19.


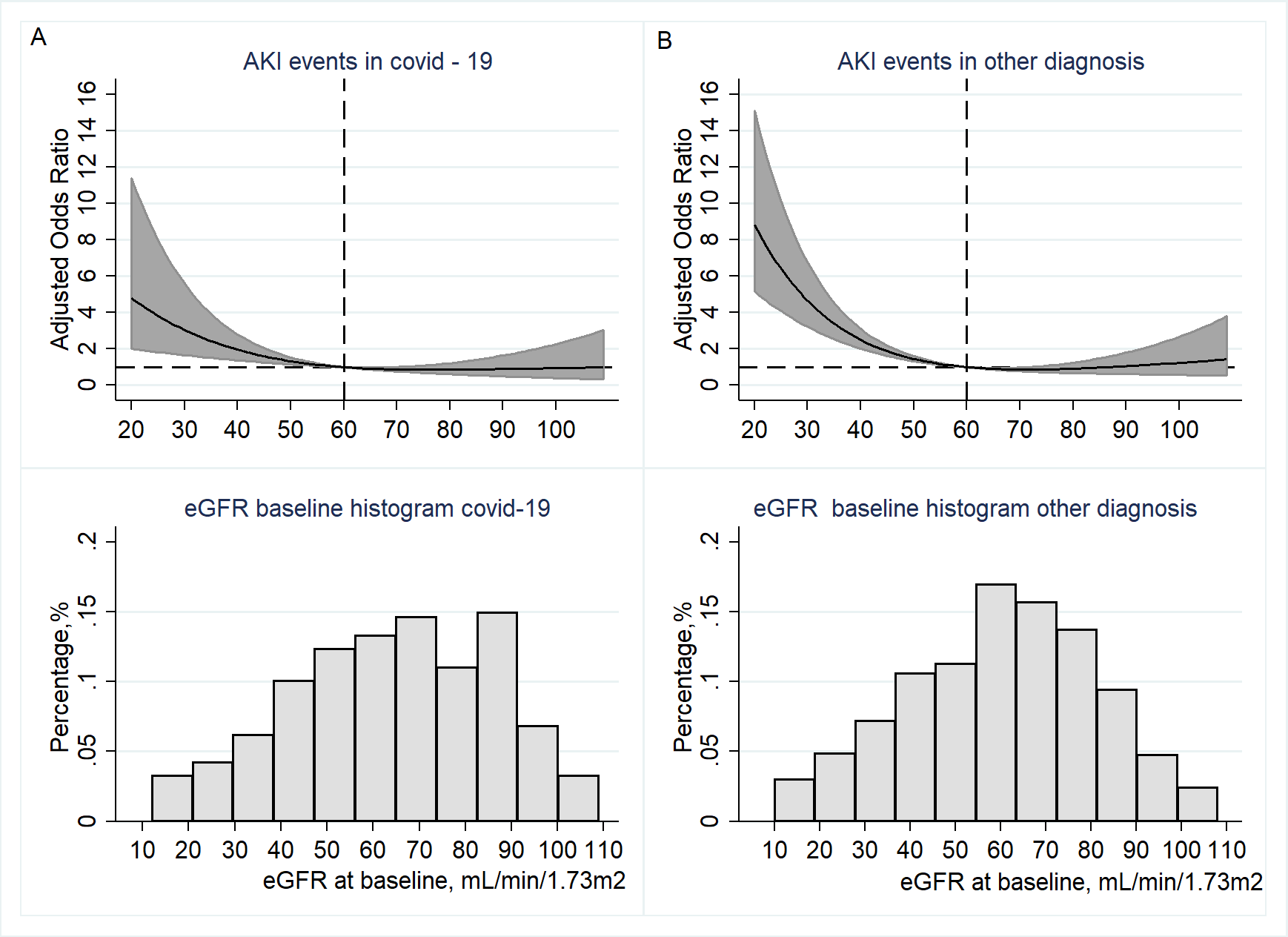
**Supplemental Figure S3.** Odds ratios for in-hospital death by serum Creatinine change (percent increase, continuous variable) using cubic splines, in patients with (A) and without (B) COVID-19. Model adjusted for age, sex, initial vital signs (temperature, SBP, DBP, pulse rate, and saturation), CRP value, number of Cr measurements, baseline eGFR value (continuous), medication (ACEIs, ARBs, beta-blockers, CCBs, diuretics, statins, LMWH/NOAC/warfarin, anti-platelets, NSAIDS, glucocorticoids and antibiotics), admission sources, and admission hospital. Data were reported as odds ratios (OR) and 95% confidence intervals (CI). In the lower part, percentage of participants across serum Creatinine change.


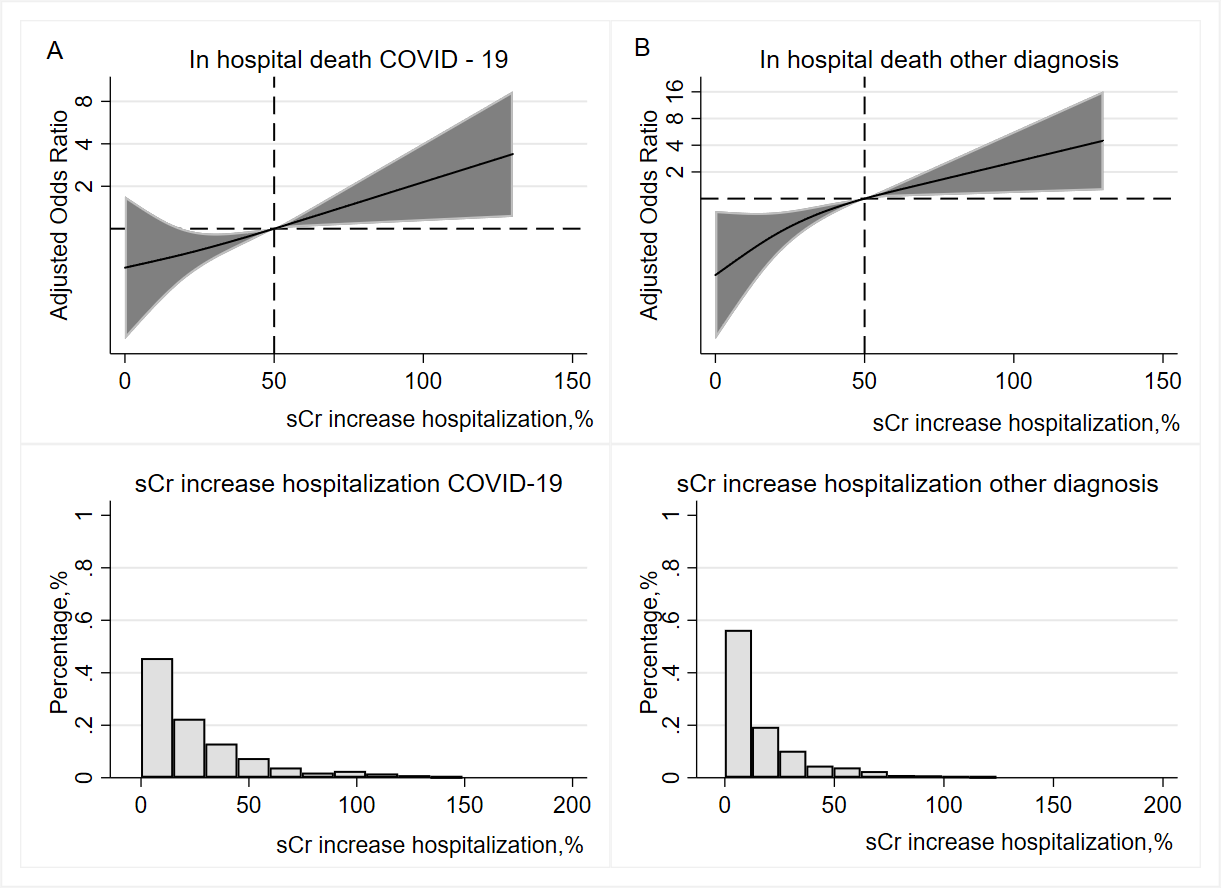

Supplement: Supplementary file 1 — Supplementary file1 (DOCX 291 KB) [file 40620_2021_1022_MOESM1_ESM.docx]
